# Washington L. Atlee and the Revival of Ovariotomy

**Published:** 1885-03

**Authors:** 


### EDITORIAL.

It is a matter of regret that it becomes necessary to announce that Dr. James Nevins Hyde, the senior editor of the JOURNAL AND EXAMINER, has found that the numerous other demands upon his time have compelled him to relinquish his editorial work. This regret will be shared by the readers of the JOURNAL AND EXAMINER, who, for so many years have been favored with his editorials. It is, however, gratifying to be enabled to assure our readers that, from time to time, they will still be favored with an article from his pen, one of which forms the leading article of this month's issue.

WASHINGTON L. ATLEE AND THE REVIVAL OF OVARIOTOMY.

Sir Spencer Wells, Bart., has recently delivered a lecture, before the Midland Medical Society, on "The Revival of Ovariotomy and its Influence on Modern Surgery." While admitting that Ephraim McDowell, of Danville, Ky., was the first ovariotomist, Sir Spencer Wells claims the honor of naturalizing the operation.

Dr. Thomas Addis Emmet acknowledges the service of the great English surgeon as paramount to all others "in popularizing the operation and making its details so familiar."

An editorial appeared in the columns of the *Medical Record*, of New York, February 21st, 1885, which alludes to the revival of ovariotomy, in the following terms:

"The theory of the operation seemed rational in conception, but disproved by results attained. There was no one to champion the cause, eliminate its errors, and bring victory out of seeming defeat. In 1858, Sir Spencer Wells set out on his mission of rendering ovariotomy a justifiable surgical expedient."

These anachronistic improprieties may be explained by the notable omission of the study of the history of medicine in the *curricula* of both English and American medical schools. While it is difficult to overlook the insularity of an Englishman, it is impossible to pardon the ignorance of an American.

Chrysmar (1819), Nathan Smith (1821), Lizars (1823), Granville (1827), Ritter (1832), Quittenbaum (1834), Stilling (1841), Charles Clay (1842), contributed to our knowledge of conditions, indications, and operative *technique*.

Dr. Washington L. Atlee, of Pennsylvania, however, deserves the credit of enfranchising the operation. The period of Dr. Atlee's activity dates from 1843. The publication, in 1855, of Dr. Atlee's first thirty cases marks an era in the history of the operation. The prize essay of Dr. G. H. Lyman, of Boston, published in the following year, materially strengthened Dr. Atlee's position.

Operators and writers multiplied with astonishing rapidity, after the publication of Dr. Atlee's first thirty cases. It is foreign to our purpose to trace the history of ovariotomy until 1858.

The Germans, with characteristic justness, ascribe to Atlee the position, which Sir Spencer Wells has arrogated.

Professor Carl Braun, one of the most critical and accurate bibliographers of the day, writes in his *Lehrbuch der gesammten Gynækologie*, *Wien*, *1881*, p. 936: "W. Atlee performed it (ovariotomy) two hundred and forty-six times, from 1843 to 1871, and by this has contributed the most to its enfranchisement."

